# Disability-adjusted life years of clonorchiasis in China: a high-resolution spatial analysis

**DOI:** 10.1186/s40249-025-01396-4

**Published:** 2025-12-24

**Authors:** Men-Bao Qian, Li Wang, Ji-Lei Huang, Chang-Hai Zhou, Ting-Jun Zhu, Xiao-Nong Zhou, Ying-Si Lai, Shi-Zhu Li

**Affiliations:** 1 National Institute of Parasitic Diseases, Chinese Center for Disease Control and Prevention (Chinese Center for Tropical Diseases Research), Shanghai, China; 2 National Health Commission Key Laboratory of Parasite and Vector Biology, Shanghai, China; 3 WHO Collaborating Center for Tropical Diseases, Shanghai, China; 4 National Center for International Research On Tropical Diseases, Shanghai, China; 5 School of Global Health, Chinese Center for Tropical Diseases Research, Shanghai Jiao Tong University School of Medicine, Shanghai, China; 6 Department of Medical Statistics, School of Public Health, Sun Yat-sen University, Guangzhou, Guangdong China

**Keywords:** Clonorchiasis, Disability-adjusted life years, Bayesian geostatistical model, Disease burden, China

## Abstract

**Background:**

Clonorchiasis is caused by the ingestion of raw freshwater fish containing infective metacercariae of *Clonorchis sinensis*. This study aimed to fully evaluate disease burden in terms of disability-adjusted life years (DALYs) for clonorchiasis in China.

**Methods:**

Following our previous study which established the fine-scale prevalence distribution of *C. sinensis* infection in China, we further adopted Bayesian geostatistical models to estimate the infection intensity in terms of eggs per gram of feces (EPG) in infected individuals based on the national surveillance data of clonorchiasis between 2016 and 2021. Disability weight was then captured through its quantitative association with EPG, and used to estimate years of life living with a disability (YLDs). Incidence of cholangiocarcinoma attributed to *C. sinensis* infection was employed to calculate years of life lost (YLLs). DALYs was then estimated at 5 × 5 km^2^ resolution, and aggregated by areas and populations.

**Results:**

In 2020, 431,009 [95% Bayesian credible interval (BCI): 370,427 to 500,553] DALYs were exerted due to clonorchiasis in China, of which 372,918 (95% BCI: 318,775–435,727) was due to YLDs and 57,998 (95% BCI: 50,816–66,069) due to YLLs. The DALYs, YLDs and YLLs per 1000 were 0.31 (95% BCI: 0.26–0.35), 0.26 (95% BCI: 0.23–0.31), and 0.04 (95% BCI: 0.04–0.05), respectively. The DALYs predominantly distributed in southern areas including Guangxi (201,029, 95% BCI: 157,589–248,287) and Guangdong (161,958, 95% BCI: 128,326–211,358). The DALYs was over doubled in male (302,678, 95% BCI: 262,028–348,300) than in female (127,970, 95% BCI: 106,834–151,699), and high in middle aged population.

**Conclusions:**

Clonorchiasis causes significant disease burden in China especially in southern areas including Guangxi and Guangdong. Urgent control is needed for clonorchiasis in the endemic areas with high burden, and adult males need to be prioritized.

**Graphical Abstract:**

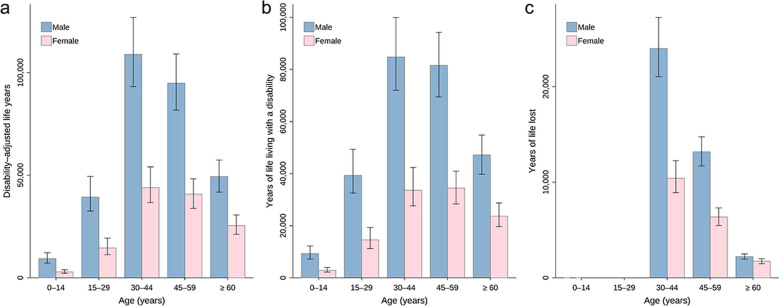

**Supplementary Information:**

The online version contains supplementary material available at 10.1186/s40249-025-01396-4.

## Background

Clonorchiasis ranks among the food-borne trematodiases. The habit of consuming raw freshwater fish causes the endemicity of clonorchiasis in eastern Asia, including China, the Republic of Korea, northern Vietnam and far east of Russia [1, 2]. Adult worms parasitize in liver of human beings, and thus lead to diverse hepatobiliary morbidities, e.g. gallstone, cholecystitis, and cholangitis [3–6]. In particular, *Clonorchis sinensis* is a definite carcinogen, leading to fatal cholangiocarcinoma [7]. These damages are highly dependent on the worm burden, which is usually indicated by the number of eggs in feces, e.g., eggs per gram of feces (EPG) [4, 6].

As one important neglected tropical disease, epidemiological data for clonorchiasis is scarce. In a previous study, we established Bayesian geostatistical models to capture the fine-scale distribution of clonorchiasis in China, based on the national surveillance data between 2016 and 2021 [8]. In 2020, a population-weighted prevalence of 0.67% was estimated, corresponding to 9.46 million persons under infection in China [8]. To fully evaluate the disease burden, not only the number of infection cases but also the intensity of infection needs to be considered. Disability-adjusted life years (DALYs) is the most comprehensive indicator to measure disease burden, which is consisted of the years of life living with a disability (YLDs) and years of life lost (YLLs) [9, 10]. Although many neglected tropical diseases (NTDs) have been included in the Global Burden of Diseases (GBD) 2021 including food-borne trematodiases (clonorchiasis, opisthorchiasis, fascioliasis, intestinal flukes, and paragonimiasis), the burden of clonorchiasis was not presented separately [11].

In this study, we aimed to establish the fine-scale distribution of *C. sinensis* infection intensity in China (5 × 5 km^2^) using Bayesian geostatistical models, and then integrate it into the framework of DALYs to estimate the high-resolution distribution of disease burden and analyze the distribution characteristics in space and population.

## Methods

### Epidemiological data

In brief, age- and gender-specific prevalence and intensity (in terms of EPG) of *C. sinensis* infection at village/community level and the behavior of ingesting raw freshwater fish locally between 2016 and 2021 was retrieved from the National Institute of Parasitic Diseases, Chinese Center for Disease Control and Prevention (Chinese Center for Tropical Diseases Research) [8]. Population data with age and gender structures between 2016 and 2020 sourced from Worldpop [12].

The probability of local practice of ingesting raw freshwater fish with a spatial resolution of 5 × 5 km^2^ referred to our previous study, in which Bayesian geostatistical logistic regression model was adopted using the behavior of ingesting raw freshwater fish locally between 2016 and 2021 in China [8]. The fine-scale distribution of *C. sinensis* prevalence in China with a spatial resolution of 5 × 5 km^2^ also referred to the previous study, in which Bayesian logistic regression model was used based on the surveillance data between 2016 and 2021, together with socioeconomic, environmental and behavioral determinants [8].

### Statistical analysis

The participants in each village/community were divided into five age groups for both genders, namely 0–14, 15–29, 30–44, 45–59 and over 60 years old. The value of EPG was calculated through multiplying the average number of eggs in two smears by a coefficient of 24, which was further summarized to average EPG for each age and gender group, by calculating the arithmetic mean of EPG values of the individuals infected with *C. sinensis*.

Both Bayesian linear model and Bayesian hurdle model for log(EPG) was applied, the latter of which considered the zero-inflated issue of the survey data (Supplementary Method S1 and Supplementary Method S2) [13, 14]. The model with better model performance was then adopted. To be noted, *C. sinensis* infection intensity is highly relevant to the prevalence, the practice of ingesting raw freshwater fish, population characteristics (including gender and ages) [15, 16], which were thus included as covariates in the models. The model performance was evaluated through five-fold cross-validation, and the one with the lowest mean absolute error (MAE) was selected as the optimal one, while other evaluation metrics, including mean error and the proportion of Bayesian credible interval (BCI) covering observed values were also considered to comprehensively assess the model’s performance. *C. sinensis* infection intensity was estimated on a regular grid covering 557,549 pixels across China, with a spatial resolution of 5 × 5 km^2^. The pixel-based age- and gender-specific estimations were first established, and then, by projecting to the gridded population, the population-adjusted intensity was estimated, either at pixel-level or aggregated administrative levels (e.g., county, provincial and national level).

DALYs were calculated following the formulas as followings:

DALYs = YLLs + YLDs;

YLDs = YLDs_1_ + YLDs_2_;

YLLs = Population × Prevalence of *C. sinensis* × Incidence of cholangiocarcinoma attributed to *C. sinensis* infection × (Life expectancy－Death age due to cholangiocarcinoma); 

YLDs_1_ = Population × Incidence of *C. sinensis* × Disability weight of *C. sinensis* infection excluding cholangiocarcinoma × Duration of *C. sinensis*;

YLDs_2_ = Population × Prevalence of *C. sinensis* × Incidence of cholangiocarcinoma attributed to *C. sinensis* infection × Disability weight of cholangiocarcinoma during survival × Survival time with cholangiocarcinoma.

Disability weight of *C. sinensis* infection excluding cholangiocarcinoma was determined by EPG. The fine-scale distribution of *C. sinensis* intensity (in terms of average EPG) of the infected individuals at 5 × 5 km^2^ resolution was used to estimate the disability weight based on their quantitative relationship (Supplementary Method S3) [17].

As prevalence, not incidence, of *C. sinensis* infection was available, thus we used the quantitative relationship in which the prevalence is equal to incidence multiplied by the duration of the condition [18, 19]. In other words, the duration of *C. sinensis* was set to 1 year in such case. Because cholangiocarcinoma demonstrates a poor prognosis with most cases experiencing fatality within 1 year, the survival time for it was fixed to 1 year [20, 21]. The age presented with cholangiocarcinoma within each age group was defined as the mean age of the infected individuals in surveillance cases in each province corresponding to that age group. Therefore, the death age of cholangiocarcinoma was calculated by adding 1 year to the age presented with cholangiocarcinoma, considering the 1-year survival period for cholangiocarcinoma. While cholangiocarcinoma tends to progress chronically, over 80% of infected individuals aged over 30 reported a history of consuming raw freshwater fish for more than 5 years [15]. Consequently, this study exclusively focused on YLLs for those aged over 30, and corresponding YLDs during survival with cholangiocarcinoma. The cholangiocarcinoma incidence attributed to *C. sinensis* infection is 35/100,000 and 25/100,000 in male and female, respectively [22]. The life expectancy in China was set as 74.52 years in male and 79.92 years in female [23]. Disability weight of the individuals with cholangiocarcinoma was set as 0.264 [24].

To identify local spatial clusters of clonorchiasis burden, we applied Local Moran’s *I* statistic at the county level. This method detects whether a county’s disease burden is significantly similar to (High-High or Low-Low) or dissimilar from (High-Low or Low-High) its neighboring counties, beyond what would be expected under spatial randomness [25]. We constructed a queen-contiguity spatial weights matrix (counties sharing a boundary or vertex are neighbors) and assessed statistical significance at *P* < 0.05. Results were classified into five categories: High-High, Low-Low, High-Low, Low-High, and Not Significant.

Statistical software R 4.2.3 (Lucent Technologies, Jasmine Mountain, USA) was utilized for statistical analysis, and the Bayesian geostatistical models were implemented using INLA package [14]. The maps were created using ArcGIS 10.1 (ESRI, Redlands, CA, USA).

## Results

### Epidemiological profiles of observed data in the surveillance spots

During 2016 and 2021, out of 1,873,668 participants from 31 provincial-level administrative divisions (PLADs), 18,550 were detected with *C. sinensis* infection, yielding a crude prevalence of 0.99%. *C. sinensis* cases were detected in 19 PLADs, and the crude average of EPG at national level was 1200 (Supplementary Table S1). EPG was higher in male (1355) than in female (913) and in elder people (Supplementary Table S2).

### Performance of models

We selected the hurdle model with better predictive performance to estimate the nationwide EPG (Supplementary Figure S1 and Supplementary Figure S2). The MAE was superior in the hurdle model with a lower value compared to linear model (481.30 vs 518.12), and the 95% coverage was higher in the former than in the latter (61.71% vs 15.24%). In the Bayesian geostatistical hurdle model for *C. sinensis* infection intensity, the posterior median of the coefficient for *C. sinensis* prevalence was 0.31 (95% BCI: 0.26–0.36), for the probability of local practice of ingesting raw freshwater fish 0.25 (95% BCI: 0.11–0.39), and for male gender 0.33 (95% BCI: 0.26–0.40). Compared to the 0–14 age group, the posterior medians for age groups 15–29, 30–44, 45–59, and ≥ 60 years were 0.08 (95% BCI: −0.07–0.22), 0.40 (95% BCI: 0.26–0.53), 0.55 (95% BCI: 0.41–0.68), and 0.52 (95% BCI: 0.39–0.66), respectively (Supplementary Table S3).

### National infection intensity of clonorchiasis

The national average of estimated EPG was 382 (95% BCI: 348–419), but at provincial level the number was overall relatively low in those with high prevalence (Supplementary Table S1 and Supplementary Figure S3). The average of estimated EPG was higher in male (435, 95% BCI: 391–484) than in female (283, 95% BCI: 253–318) (Supplementary Table S2 and Supplementary Figure S4). The average of estimated EPG was 212 (95% BCI: 178–266), 250 (95% BCI: 217–304), 386 (95% BCI: 334–452), 453 (95% BCI: 401–534), and 436 (95% BCI: 378–502) in age groups 0–14, 15–29, 30–44, 45–59, and ≥ 60 years, respectively (Supplementary Table S2 and Supplementary Figure S4).

### Disability-adjusted life years of clonorchiasis

**Fig. 1:**
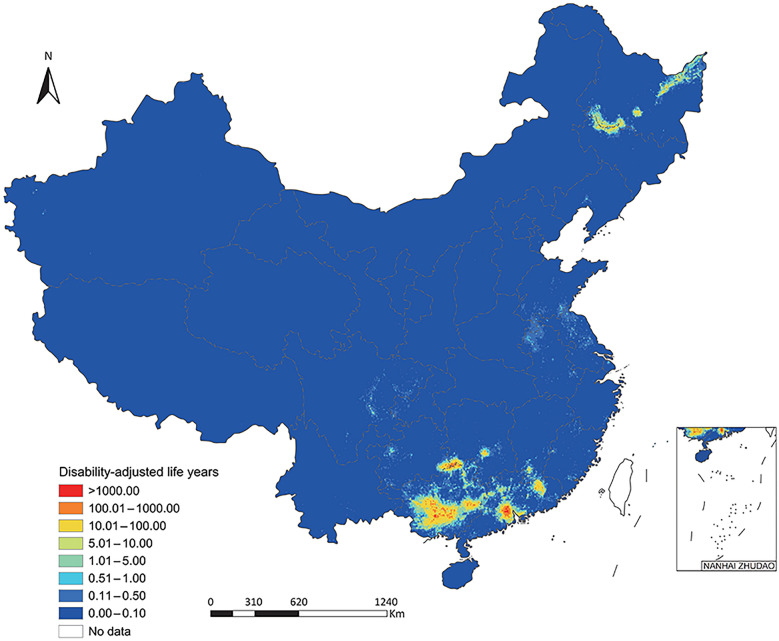
Disability-adjusted life years of *Clonorchis sinensis* infection in China at 5 × 5 km^2^ resolution. Map approval number: GS(2025)5958

**Fig. 2:**
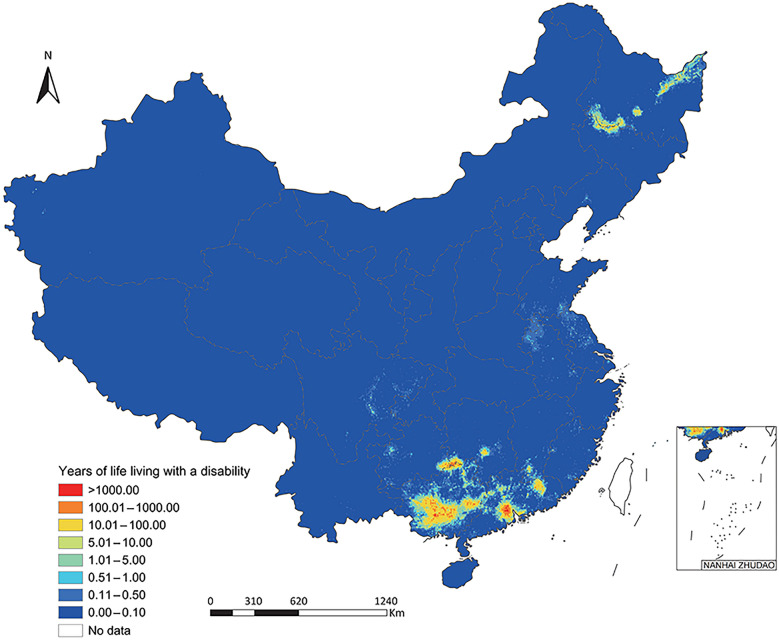
Years of life living with a disability of *Clonorchis sinensis* infection in China at 5 × 5 km^2^ resolution. GS(2025)5958

**Fig. 3:**
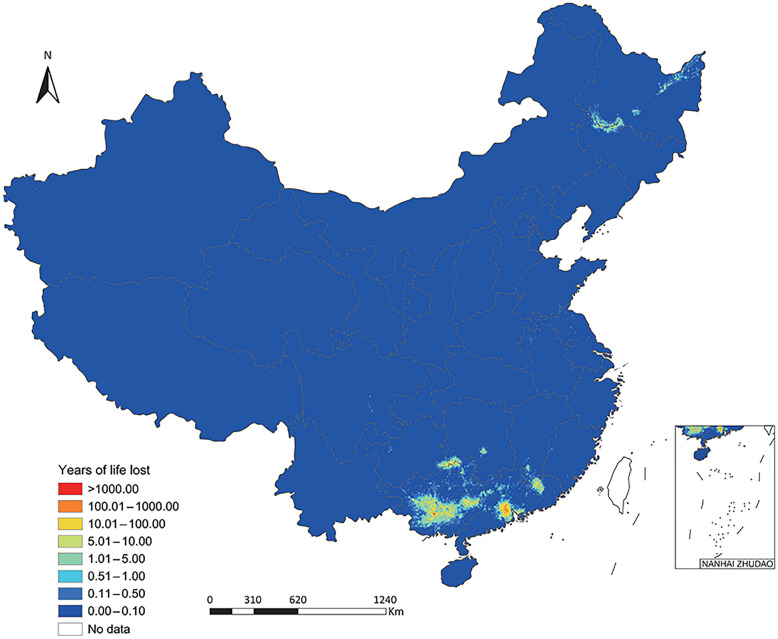
Years of life lost of *Clonorchis sinensis* infection in China at 5 × 5 km^2^ resolution. GS(2025)5958

**Fig. 4:**
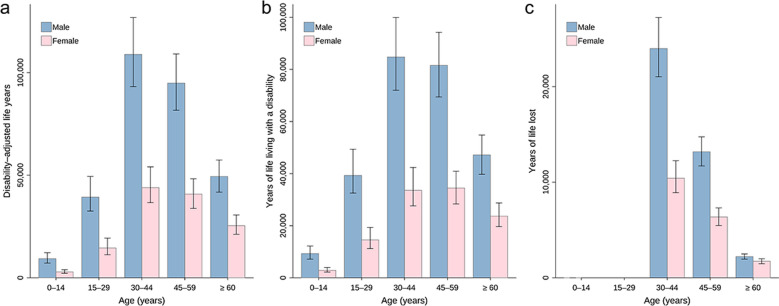
Disease burden of *Clonorchis sinensis* infection by genders and age groups in China

Totally, 431,009 (95% BCI: 370,427–500,553) DALYs were exerted, of which 372,918 (95% BCI: 318,775–435,727) was due to YLDs and 57,998 (95% BCI: 50,816–66,069) due to YLLs (Table 1). The DALYs, YLDs and YLLs per 1000 were 0.31 (95% BCI: 0.26–0.35), 0.26 (95% BCI: 0.23–0.31), and 0.04 (95% BCI: 0.04–0.05), respectively (Table 1). The DALYs predominantly distributed in southern areas including Guangxi (201,029, 95% BCI: 157,589–248,287) and Guangdong (161,958, 95% BCI: 128,326–211,358), as well as neighboring Guizhou (7352, 95% BCI: 4503–11,469) and Hunan (6597, 95% BCI: 4887–9419) (Table 1**, **Fig. 1**, **Fig. 2, and Fig. 3). High burden was also caused in northeastern areas including Heilongjiang (23,790, 95% BCI: 18,824–30,283) and Jilin (6564, 95% BCI: 4808–9193) (Table 1**, **Fig. 1**, **Fig. 2, and Fig. 3). The DALYs was over doubled in male (302,678, 95% BCI: 262,028–348,300) than in female (127,970, 95% BCI: 106,834–151,699), while it was 12,229 (95% BCI: 9471–16,152), 53,976 (95% BCI: 44,137–68,136), 152,928 (95% BCI: 130,113–178,881), 135,492 (95% BCI: 115,676–155,848), and 75,021 (95% BCI: 63,263–87,485) in age groups 0–14, 15–29, 30–44, 45–59, and ≥ 60 years, respectively **(**Table 2**, **Fig. 4).

**Table 1:** Disease burden in terms of DALYs of *Clonorchis sinensis* infection by PLADs in China

PLADs	DALYs (median and 95% BCI)	DALYs/1000 (median and 95% BCI)	YLDs (median and 95% BCI)	YLDs/1000 (median and 95% BCI)	YLLs (median and 95% BCI)	YLLs/1000 (median and 95% BCI)
Anhui	1435.76 (797.23–2959.61)	0.02 (0.01–0.05)	1156.50 (629.42–2356.53)	0.02 (0.01–0.04)	285.09 (171.90–554.45)	0.00 (0.00–0.01)
Beijing	22.06 (0.51–1726.40)	0.00 (0.00–0.06)	18.78 (0.44–1369.55)	0.00 (0.00–0.05)	3.32 (0.08–289.39)	0.00 (0.00–0.01)
Chongqing	340.68 (95.68–1983.90)	0.01 (0.00–0.07)	281.87 (79.15–1656.67)	0.01 (0.00–0.06)	57.80 (16.96–321.94)	0.00 (0.00–0.01)
Fujian	733.55 (344.38–1981.22)	0.02 (0.01–0.05)	579.52 (265.00–1618.02)	0.01 (0.01–0.04)	157.32 (78.96–375.89)	0.00 (0.00–0.01)
Gansu	176.82 (31.10–1948.25)	0.01 (0.00–0.08)	144.70 (24.74–1539.44)	0.01 (0.00–0.06)	32.09 (5.94–337.04)	0.00 (0.00–0.01)
Guangdong	161,958.09 (128,326.30–211,357.83)	1.31 (1.03–1.70)	138,419.08 (108,857.89–180,804.25)	1.12 (0.88–1.46)	23,908.53 (19,545.70–29,564.10)	0.19 (0.16–0.24)
Guangxi	201,028.94 (157,589.36–248,286.59)	4.26 (3.34–5.27)	177,011.27 (138,011.28–220,030.29)	3.75 (2.93–4.67)	24,169.69 (19,467.33–28,912.76)	0.51 (0.41–0.61)
Guizhou	7352.15 (4502.90–11,469.10)	0.22 (0.13–0.34)	6644.29 (4010.60–10,412.23)	0.20 (0.12–0.31)	690.87 (472.03–1067.67)	0.02 (0.01–0.03)
Hainan	70.89 (4.86–2357.00)	0.01 (0.00–0.23)	59.76 (4.09–2036.39)	0.01 (0.00–0.20)	11.55 (0.81–338.74)	0.00 (0.00–0.03)
Hebei	307.34 (49.95–4209.00)	0.00 (0.00–0.06)	260.50 (42.25–3591.56)	0.00 (0.00–0.05)	46.84 (7.48–578.85)	0.00 (0.00–0.01)
Heilongjiang	23,790.12 (18,824.05–30,283.21)	0.60 (0.48–0.77)	20,372.54 (16,060.73–26,040.99)	0.52 (0.41–0.66)	3374.77 (2733.40–4250.16)	0.09 (0.07–0.11)
Henan	753.72 (233.05–4973.29)	0.01 (0.00–0.05)	657.62 (202.46–4136.21)	0.01 (0.00–0.04)	101.94 (30.57–698.57)	0.00 (0.00–0.01)
Hubei	455.73 (84.70–4270.20)	0.01 (0.00–0.08)	386.39 (70.14–3707.70)	0.01 (0.00–0.07)	73.23 (13.27–637.35)	0.00 (0.00–0.01)
Hunan	6596.82 (4886.56–9418.91)	0.10 (0.07–0.14)	5910.03 (4375.63–8439.03)	0.09 (0.07–0.13)	677.50 (500.74–968.52)	0.01 (0.01–0.01)
Jiangsu	1657.62 (787.45–5402.88)	0.02 (0.01–0.07)	1334.82 (619.53–4364.20)	0.02 (0.01–0.05)	341.62 (159.35–932.10)	0.00 (0.00–0.01)
Jiangxi	2419.14 (1030.13–7583.52)	0.05 (0.02–0.16)	1893.60 (780.97–5892.74)	0.04 (0.02–0.12)	537.21 (222.61–1529.34)	0.01 (0.00–0.03)
Jilin	6563.62 (4808.07–9193.01)	0.24 (0.18–0.34)	5245.90 (3829.80–7379.47)	0.19 (0.14–0.27)	1289.40 (948.84–1790.62)	0.05 (0.03–0.07)
Liaoning	403.63 (121.76–2723.36)	0.01 (0.00–0.06)	354.37 (107.72–2358.88)	0.01 (0.00–0.05)	50.86 (13.50–360.77)	0.00 (0.00–0.01)
Neimenggu	386.27 (100.92–3378.97)	0.01 (0.00–0.13)	318.79 (81.68–2852.77)	0.01 (0.00–0.11)	67.22 (18.67–533.98)	0.00 (0.00–0.02)
Ningxia	15.45 (0.92–793.27)	0.00 (0.00–0.11)	12.93 (0.75–667.56)	0.00 (0.00–0.09)	2.48 (0.16–129.94)	0.00 (0.00–0.02)
Qinghai	12.08 (1.09–407.25)	0.00 (0.00–0.06)	9.91 (0.90–331.79)	0.00 (0.00–0.05)	2.18 (0.18–71.96)	0.00 (0.00–0.01)
Shaanxi	270.23 (30.82–3252.25)	0.01 (0.00–0.08)	229.12 (25.78–2734.34)	0.01 (0.00–0.07)	42.96 (4.77–480.81)	0.00 (0.00–0.01)
Shandong	1441.01 (516.04–5923.72)	0.01 (0.01–0.06)	1170.10 (416.44–5091.20)	0.01 (0.00–0.05)	262.98 (101.01–1030.44)	0.00 (0.00–0.01)
Shanghai	49.93 (1.28–1737.94)	0.00 (0.00–0.06)	42.98 (1.10–1509.31)	0.00 (0.00–0.05)	7.30 (0.19–235.31)	0.00 (0.00–0.01)
Shanxi	196.78 (29.67–2887.66)	0.01 (0.00–0.08)	166.12 (25.50–2546.25)	0.00 (0.00–0.07)	28.78 (4.30–421.01)	0.00 (0.00–0.01)
Sichuan	1893.18 (1004.47–4288.99)	0.02 (0.01–0.05)	1592.00 (840.17–3485.28)	0.02 (0.01–0.04)	301.00 (161.55–634.75)	0.00 (0.00–0.01)
Tianjin	21.56 (0.34–570.75)	0.00 (0.00–0.03)	18.06 (0.29–477.03)	0.00 (0.00–0.03)	3.31 (0.05–91.21)	0.00 (0.00–0.01)
Xinjiang	456.92 (147.66–2402.59)	0.02 (0.01–0.09)	372.08 (117.84–2048.57)	0.01 (0.00–0.08)	85.08 (29.00–390.20)	0.00 (0.00–0.02)
Xizang	38.16 (2.56–1028.27)	0.01 (0.00–0.22)	32.10 (2.11–861.98)	0.01 (0.00–0.19)	6.31 (0.44–143.69)	0.00 (0.00–0.03)
Yunnan	881.57 (249.70–4265.74)	0.02 (0.01–0.09)	757.07 (213.79–3692.96)	0.02 (0.00–0.08)	125.18 (36.98–576.34)	0.00 (0.00–0.01)
Zhejiang	365.75 (55.89–3237.24)	0.01 (0.00–0.05)	309.87 (46.70–2824.75)	0.00 (0.00–0.04)	55.73 (9.00–459.42)	0.00 (0.00–0.01)
Total	431,008.60 (370,426.90–500,552.64)	0.31 (0.26–0.35)	372,918.29 (318,774.76–435,726.67)	0.26 (0.23–0.31)	57,998.35 (50,816.15–66,068.89)	0.04 (0.04–0.05)

*PLADs:* provincial-level administrative divisions; *BCI:* Bayesian credible interval; *DALYs:* disability-adjusted life years; *YLDs:* years of life living with a disability; *YLLs:* years of life lost

**Table 2:** Disease burden in terms of DALYs of *Clonorchis sinensis* infection by genders and age groups in China

Characteristics	DALYs (median and 95% BCI)	DALYs/1000 (median and 95% BCI)	YLDs (median and 95% BCI)	YLDs/1000 (median and 95% BCI)	YLLs (median and 95% BCI)	YLLs/1000 (median and 95% BCI)
Male						
0–14 years	9304.96 (7185.09–12,207.62)	0.07 (0.05–0.09)	9304.96 (7185.09–12,207.62)	0.07 (0.05–0.09)	0.00 (0.00–0.00)	0.00 (0.00–0.00)
15–29 years	39,321.87 (32,521.43–49,372.77)	0.29 (0.24–0.37)	39,321.87 (32,521.43–49,372.77)	0.29 (0.24–0.37)	0.00 (0.00–0.00)	0.00 (0.00–0.00)
30–44 years	108,862.80 (93,194.99–126,854.68)	0.65 (0.56–0.76)	84,770.49 (71,997.78–99,942.31)	0.51 (0.43–0.60)	23,984.99 (21,025.33–27,226.80)	0.14 (0.13–0.16)
45–59 years	94,880.83 (81,654.19–109,096.17)	0.56 (0.48–0.64)	81,550.58 (69,429.91–94,257.16)	0.48 (0.41–0.55)	13,180.76 (11,699.20–14,745.50)	0.08 (0.07–0.09)
60 + years	49,362.57 (41,768.66–57,390.33)	0.41 (0.35–0.48)	47,185.82 (39,751.63–54,819.16)	0.39 (0.33–0.46)	2216.32 (1954.79–2493.75)	0.02 (0.02–0.02)
Subtotal	302,678.42 (262,028.19–348,300.16)	0.42 (0.36–0.48)	263,087.99 (226,236.71–303,741.56)	0.36 (0.31–0.42)	39,411.28 (34,956.98–44,557.62)	0.05 (0.05–0.06)
Female						
0–14 years	2870.99 (2192.15–3935.38)	0.03 (0.02–0.04)	2870.99 (2192.15–3935.38)	0.03 (0.02–0.04)	0.00 (0.00–0.00)	0.00 (0.00–0.00)
15–29 years	14,535.53 (11,216.88–19,336.49)	0.11 (0.09–0.15)	14,535.53 (11,216.88–19,336.49)	0.11 (0.09–0.15)	0.00 (0.00–0.00)	0.00 (0.00–0.00)
30–44 years	43,942.73 (36,628.54–54,056.88)	0.28 (0.23–0.34)	33,578.90 (27,602.24–42,375.63)	0.21 (0.17–0.27)	10,416.86 (8902.50–12,248.68)	0.07 (0.06–0.08)
45–59 years	40,797.00 (33,819.67–48,245.63)	0.25 (0.21–0.29)	34,476.70 (28,340.99–40,923.16)	0.21 (0.17–0.25)	6363.89 (5470.53–7313.84)	0.04 (0.03–0.04)
60 + years	25,423.81 (21,202.44–30,625.45)	0.20 (0.17–0.25)	23,688.40 (19,643.49–28,733.61)	0.19 (0.16–0.23)	1729.99 (1473.27–1987.52)	0.01 (0.01–0.02)
Subtotal	127,969.61 (106,833.76–151,699.08)	0.19 (0.16–0.22)	109,440.69 (91,154.52–130,144.05)	0.16 (0.13–0.19)	18,542.44 (15,859.17–21,563.49)	0.03 (0.02–0.03)
Total	431,008.60 (370,426.90–500,552.64)	0.31 (0.26–0.35)	372,918.29 (318,774.76–435,726.67)	0.26 (0.23–0.31)	57,998.35 (50,816.15–66,068.89)	0.04 (0.04–0.05)

*BCI:* Bayesian credible interval; *DALYs:* disability-adjusted life years; *YLDs:* years of life living with a disability; *YLLs:* years of life lost

### Spatial clustering of clonorchiasis burden

Local Moran’s *I* analysis revealed significant spatial autocorrelation in county-level DALYs (per 1000 persons). High-High clusters—indicating counties with high disease burden surrounded by other high-burden counties—were predominantly concentrated in Guangxi, southern Guangdong, and border areas among Guangxi, Hunan and Guizhou. Additional High-High clusters were observed in border areas along southwestern Heilongjiang and northwestern Jilin, and northeastern Heilongjiang. A small number of Low-High anomalies—counties with low burden surrounded by high-burden neighbors—were identified, primarily along the borders of Guangxi and Guangdong, Guangxi and Hunan, Guangxi and Guizhou. The majority of counties showed no significant spatial autocorrelation (Supplementary Figure S5).

## Discussion

This study illuminates a high burden of clonorchiasis in China, indicated by the DALYs. The high DALYs is caused by both the high prevalence [8] and intensity of infection. Although YLLs are also high, YLDs dominate the components of DALYs, which shows the source of burden in clonorchiasis from morbidity other than mortality. The distribution of clonorchiasis burden shows significant variation among areas and populations, with high DALYs in southern China including Guangxi and Guangdong, and adult males.

Corresponding to the inadequate studies for clonorchiasis, researches on disease burden in terms of DALYs are extremely scarce [26]. However, this indicator is of crucial importance, because clonorchiasis predominantly causes loss due to morbidity other than mortality, as demonstrated in this study. Thus, it is necessary to comprehensively consider the burden exerted by clonorchiasis in population. By now, a few studies estimated the DALYs of clonorchiasis [27]. A figure of 231,547 DALYs in China was estimated in 2005, while 12.91 million persons were estimated under infection with *C. sinensis* [27]. Only the heavy infection with an EPG over 10,000 was considered, when the corresponding disability weight was fixed to 0.104. This strategy has also been employed in GBD 2021 [28]. On one hand, light and moderate infections dominate the infection spectrum and they also cause significant loss of health [4, 29]. On the other hand, differential disability weight should be employed for different intensity of infection [17].

The symptoms and morbidities of *C. sinensis* infection is relevant positively to the worm burden which is usually indicated by the EPG [4, 6, 30]. Especially, the quantitative relationship between them captured by our team paves the way for accurate estimation of burden for clonorchiasis [17]. The findings justify the importance to consider both infection and intensity in evaluation of disease burden. For example, the prevalence of *C. sinensis* infection was 0.85% in male and 0.48% in female, leading to a ratio of 1.77 [8], while the corresponding EPG was 435 and 283 with a ratio of 1.53, which thus leads to a higher ratio in the DALYs between them (2.37 times, 302,678 vs 127,970). Thus, DALYs considering both prevalence and intensity could comprehensively demonstrate the burden of clonorchiasis.

Although geostatistical model has been widely applied to map the epidemiology of helminthiasis, most studies focus on prevalence or infection, while few studies on intensity of infection [31–33]. This study demonstrates the feasibility and value of this technique in mapping intensity of infection. In the selection of model covariates, four indicators were considered, namely the prevalence of *C. sinensis* infection, the probability of local practice of ingesting raw freshwater fish, age groups, and gender. Not surprisingly, they were all finally included in the model. Infection intensity is determined by the practice of ingesting raw freshwater fish, including the frequency and quantity, both of which are relative to gender and ages [15, 34]. In the prevalence estimation, many environmental and socioeconomic factors have already been included and thus they were not considered in infection intensity estimation to avoid the collinearity [8]. It is to be emphasized that the overall consistence in high prevalence, heavy EPG of the infected individuals and existence of the practice of ingesting raw freshwater fish. However, the estimated EPG was also found high in some areas with low prevalence. Indeed, in the corresponding surveillance villages/communities, only several cases with high EPG were detected, which lead to the low prevalence but high average EPG. It was argued that these cases probably consumed raw freshwater fish in high prevalent areas during their trip or earlier living there [35, 36].

Clonorchisis is becoming a severe neglected tropical disease and thus an important public health problem in China. Compared to the significant control and even elimination of many other NTDs [37, 38], the endemicity of clonorchiasis persists [39]. Social-economic development benefits the control of most NTDs excluding clonorchiasis, because more and more people could afford to ingest raw freshwater fish. Indeed, the highest burden of clonorchiasis in Guangxi is relevant to recent increasing endemicity there [29]. Thus, the adoption of intensified control is urgent [40, 41]. Because of the high prevalence and intensity of infection, morbidity control should be set as the main aim currently, in which chemotherapy is one important pillar to decrease the morbidity and corresponding DALYs [23, 42, 43]. Spatial clustering analysis reveals the dual geographic epicenters of burden in southern and northeastern China, which need to be prioritized. Of course, health education to change the special habit of ingesting raw freshwater fish and environmental modification to block the contamination of feces into water should also be integrated to increase the sustainability [2].

This study has several limitations. First, the estimated burden in our study might be still underestimated, as the disability weight deduced form the EPG doesn’t consider all damages exerted by clonorchiasis [17]. Additionally, we completely exclude the cholangiocarcinoma attributed to *C. sinensis* in those individuals aged below 30 years old. Thus, more research is expected in future. Second, infection intensity-specific disability weight estimates the burden of morbidities accurately, but the specific cholangiocarcinoma data could not be provided. Indeed, the higher incidence in male compared to female demonstrates the effect of infection intensity [22], but a group of intensity specific incidence is also expected in future. Third, as other helminths, the infection of *C. sinensis* is usually overdispersed, with a few infected individuals harboring many worms. However, we didn’t subdivide the infected individuals but used average EPG to estimate the overall disability weight. This benefits to cover the burden from those with light intensity. Forth, time trend was not presented here, because of the nonrandom sampling in surveillance and relative short period covered in this study [8]. It is expected the methodology here could be used to evaluate the changing trend of clonorchiasis burden in future.

## Conclusions

Clonorchiasis is causing high disease burden, and becoming an important public health problem in China. YLDs dominate the components of DALYs, with a proportion of over 85%. Burden of clonorchiasis shows high variation by areas and populations. Special high burden occurs in southern China, while adults especially men contribute to most burden, due to both high prevalence and intensity of infection in these areas and populations. Thus, intensified control needs to be implemented in China, especially targeting key areas and populations. Our findings also justify the reasonability to include both infection and intensity in evaluating disease burden for helminthiases.

## Supplementary Information

Supplementary Material 1

## Data Availability

Data are not publicly available but are available on reasonable request after reviewed by the National Institute of Parasitic Diseases, Chinese Center for Disease Control and Prevention (Chinese Center for Tropical Diseases Research).

## Abbreviations

BCI: Bayesian credible interval
DALYs: Disability-adjusted life years
EPG: Eggs per gram of feces
GBD: Global Burden of Diseases
MAE: Mean absolute error
NTDs: Neglected tropical diseases
PLADs: Provincial-level administrative divisions
YLDs: Years of life living with a disability
YLLs: Years of life lost
