# Supplementary material for: Disability-adjusted life years of clonorchiasis in China: a high-resolution spatial analysis
Source: Infect Dis Poverty. 2025 Dec 24;14:126. doi: 10.1186/s40249-025-01396-4 (PMC12729697; doi:10.1186/s40249-025-01396-4)
Supplement: Supplementary file 1 — Supplementary Material 1 [file 40249_2025_1396_MOESM1_ESM.docx]

**Disability-adjusted life years of clonorchiasis in China: a high-resolution spatial analysis**

Men-Bao Qian^1,2,3,4,5*†^, Li Wang^6†^ , Ji-Lei Huang^1,2,3,4^, Chang-Hai Zhou^1,2,3,4^, Ting-Jun Zhu^1,2,3,4^, Xiao-Nong Zhou^1,2,3,4,5*^, Ying-Si Lai^6*^, Shi-Zhu Li^1,2,3,4,5*^

**Supplementary Information**

[Method S1: Bayesian geostatistical linear regression model for log(EPG) 2](#_Toc212467323)

[Method S2: Bayesian geostatistical hurdle model for log(EPG) 3](#_Toc212467324)

[Method S3: Disability weight of *Clonorchis sinensis* infection 5](#_Toc212467325)

[Table S1: Observed and estimated EPG of the infected individuals with *Clonorchis sinensis* by provinces in China 7](#_Toc212467326)

[Table S2: Observed and estimated EPG of the infected individuals with *Clonorchis sinensis* by genders and age groups in China 8](#_Toc212467327)

[Table S3: Posterior summaries of the Bayesian geostatistical hurdle model parameters for *Clonorchis sinensis* intensity in term of EPG 9](#_Toc212467328)

[Figure S1: Cross validation of the Bayesian geostatistical hurdle model for log(EPG) 10](#_Toc212467329)

[Figure S2: Cross validation of the Bayesian geostatistical linear model for log(EPG) 11](#_Toc212467330)

[Figure S3: Arithmetic mean of eggs per gram feces of *Clonorchis sinensis* infection in China at 5×5 km^2^ resolution 12](#_Toc212467331)

[Figure S4: Arithmetic mean of eggs per gram feces of *Clonorchis sinensis* infection by genders and age groups in China 13](#_Toc212467332)

[Figure S5: Spatial clusters of *Clonorchis sinensis* infection at county level in China (disability–adjusted life years per 1000 persons) 14](#_Toc212467333)

[References 15](#_Toc212467334)

# Method S1: Bayesian geostatistical linear regression model for log(EPG)

For the sensitivity analysis, we also tried a Bayesian geostatistical linear model to estimate the spatial distribution of EPG [1]. The formula of the model is as follows:

$$y_{i}=log({EPG}_{i})$$

$$y_{i} \sim N(\mu_{i}, \sigma)$$

$\mu_{i}$= $\beta_{0}+\sum_{m=1}^{M} \beta_{m}x_{mi}+ u_{i}+\upsilon_{i}$

$y_{i}$ is the amount of log(EPG) at location *i*. $\beta_{0}$ is the intercept, and $\beta_{m}$ is the coefficients of the covariate $x_{m}$. $\beta_{0} \sim N(0, 1000)$, $\beta_{m} \sim N\left( 0, 1000 \right)$. $u_{i}$ and $\upsilon_{i}$ represent location-specific spatial and exchangeable random effects, respectively. $\boldsymbol{u=}\left( u_{1}, u_{2}, \ldots, u_{l} \right)^{T}$ is arising from a multivariate norm distribution $\boldsymbol{u} \sim MVN(0, \boldsymbol{\Sigma})$, where $\boldsymbol{\Sigma}$ is a Matérn covariance matrix. $\sum_{ij} = \sigma_{sp}^{2}{(\kappa d_{ij})}^{\upsilon}K_{\upsilon}(\kappa d_{ij})/(\Gamma(\upsilon)2^{\upsilon-1}).$ $d_{ij}$ is the Euclidean distance between location $i$ and $j$, $\kappa$ is a scaling parameter, $log(\kappa) \sim lognorm(0, 100)$, $\upsilon$ represents a smoothing parameter with a specific value of 1, $K_{\upsilon}$ represents the modified Bessel function of the second kind with order $\upsilon$. The spatial range $\rho= \sqrt{8}/\kappa$ is considered as the distance that spatial correlation is less than 0.1. Less-informative priors are adopted as: $log(\tau_{sp}) \sim lognormal(0, 100)$ and $log(\tau_{nonsp}) \sim logGamma(1, 0.00005)$, with $\sigma_{sp}^{2} = 1/(4\pi\kappa^{2}\tau_{sp}^{2})$ and $\upsilon_{i} \sim N(0, 1/\tau_{nonsp})$,*.*

# Method S2: Bayesian geostatistical hurdle model for log(EPG)

We constructed a Bayesian geostatistical hurdle regression model to estimate the spatial distribution of average EPG [2]. The model consists of two parts: the first part to model the occurrence of local mean EPG > 0, and the second part to model the amount of log(EPG).

For the first part,

$$z_{i}\left\{ \begin{aligned} 0, if EPG = 0 at location i \\ 1, if EPG > 0 at location i \end{aligned} \right.$$

$$z_{i} \sim Bernoulli(\pi_{iz})$$

$$logit(\pi_{iz}) = \alpha^{z} + \xi_{i}$$

For the second part,

$$y_{i}\left\{ \begin{aligned} NA, if EPG = 0 at location i \\ log({EPG}_{i}), if EPG > 0 at locatin i \end{aligned} \right.$$

$$y_{i} \sim N(\mu_{i}, \sigma^{2})$$

$$log\left( \mu_{i} \right)= \alpha^{y} + {\beta\xi}_{i} +\sum_{m=1}^{M} \beta_{m}x_{mi}+u_{i}+\upsilon_{i}$$

We choose to set a Bernoulli distribution for $z_{i}$ and Normal distribution for $y_{i}$. $\pi_{iz}$ is the probability of EPG > 0 at location $i$. $\alpha^{z}$ and $\alpha^{y}$ are the intercepts in the first part and second part, respectively, with less-informative normal distributions as their priors $\alpha^{z}, \alpha^{y} \sim N(0, 1000)$. $\beta$ is the scaling parameter for $\xi_{i}$, and a normal distribution prior is set for $\beta$, $\beta\sim N(0, 1)$. $\xi_{i}$ is the spatial effect considered for the probability of EPG > 0, which is being shared in the linear model for $log(\mathrm{EPG}_{i})$ amount. We assume that the vector ${\boldsymbol{\xi}= (\xi_{1}, \xi_{2}, \ldots, \xi_{l})}^{T}$ is arising from a multivariate normal distribution $\boldsymbol{\xi} \sim MVN(0, \boldsymbol{\Sigma}^{\boldsymbol{(1)}})$, where $\boldsymbol{\Sigma}^{\boldsymbol{(1)}}$ is a Matérn covariance matrix, $\Sigma_{ij}^{(1)}$=$\sigma_{sp1}^{2}{(\kappa_{1}d_{ij})}^{\upsilon}K_{\upsilon}(\kappa_{1}d_{ij})/(\Gamma(\upsilon)2^{\upsilon-1})$. $d_{ij}$ is the Euclidean distance between location $i$ and $j$, $\kappa_{1}$ is a scaling parameter, $\upsilon$ represents a smoothing parameter with a specific value of 1, and $K_{\upsilon}$ represents the modified Bessel function of the second kind with order $\upsilon$. The spatial range $\rho_{1} = \sqrt{8}/{\kappa_{1}}$ is considered as the distance that spatial correlation is less than 0.1. The PC priors are set for spatial range $\rho_{1}$ and marginal standard deviation $\sigma_{sp}$, $P\left( \rho_{1}<1 \right)=0.5$ and $P\left( \sigma_{sp1}>0.5 \right)=0.5$[3]. ${\boldsymbol{u}= (u_{1}, u_{2}, \ldots, u_{l})}^{T}$, another spatial random effect in the model for the amount of log(EPG), is assumed to followed a multivariate normal distribution, $\boldsymbol{u} \sim MVN(0, \boldsymbol{\Sigma}^{\boldsymbol{(2)}})$, where $\boldsymbol{\Sigma}^{\boldsymbol{(2)}}$ is a Matérn covariance matrix, $\Sigma_{ij}^{(2)}$=$\sigma_{sp2}^{2}{(\kappa_{2}d_{ij})}^{\upsilon}K_{\upsilon}(\kappa_{2}d_{ij})/(\Gamma(\upsilon)2^{\upsilon-1}).$ $d_{ij}$, $\kappa_{2}$, $\upsilon$ and $K_{\upsilon}$ have the similar meanings as represented in $\boldsymbol{\xi}$. The spatial range $\rho_{2} = \sqrt{8}/{\kappa_{2}}$ is considered as the distance that spatial correlation is less than 0.1. The PC priors are set for spatial range $\rho_{2}$ and marginal standard deviation $\sigma_{\boldsymbol{2}}$, $P\left( \rho_{2}<1 \right)=0.5$ and $P\left( \sigma_{sp2}>0.5 \right)=0.5$[3]. $\upsilon_{i}$ represents location-specific non-spatial effects with $\upsilon_{i} \sim N(0, 1/\tau_{nonsp})$. $\tau_{nonsp}=1/\sigma_{nonsp}$ is the non-spatial effect precision, with less informative prior $log(\tau_{nonsp}) \sim logGamma(1, 0.00005)$*.* $\beta_{m}$ is the regression coefficient of covariate $x_{m}$, and a less-informative normal distribution is specified for its prior $\beta_{m} \sim N\left( 0, 1000 \right)$. Less informative priors are also set for $\kappa_{1}$ and $\kappa_{2}$ as $log(\kappa_{1}) \sim lognorm(0, 100)$ and $log(\kappa_{2}) \sim lognorm(0, 100)$, respectively.

# Method S3: Disability weight of *Clonorchis sinensis* infection

Our team has captured the disability weight of *C. sinensis* infection [4]. Community-based survey was carried out to get the probability of single sequelae caused by *C. sinensis* infection, while the corresponding disability weight for the single sequelae was got through searching the literatures. Then, disability weight of *C. sinensis* infection was captured through summing the production of the probability and disability weight of all sequelae. Here we revisited the data. Beside the disability weight by age groups, the corresponding arithmetic mean of EPG was extracted (**Table A**).

**Table A. Arithmetic mean of EPG and disability weight in each age group**

| **Age groups (years)** | **Arithmetic mean of EPG** | **Disability weight** |
| --- | --- | --- |
| 5–14 | 135 | 0.022 |
| 15–29 | 417 | 0.052 |
| 30–44 | 480 | 0.072 |
| 45–59 | 2654 | 0.094 |
| 60+ | 3525 | 0.118 |

A logarithmic equation was then used to fit their relationship, as below:

*y* = 0.0263ln(*x*)-0.1027 (*R*^2^=0.9392),

where *y* stood for disability weight and *x* represented the arithmetic mean of EPG.

Finally, this equation was adopted to model the disability weight for different genders and age groups at village/community levels in this study based on their arithmetic mean of EPG.

The observed range of arithmetic mean of EPG was between 135 and 3525. The maximum of arithmetic mean of EPG in this study was 3302, falling in the range. To capture the disability weight of those below 135, we extrapolated the range. Considering the range of disability weight between 0 and 1, any value of *y* smaller than zero is set to zero. This extrapolation is reasonable. First, the extrapolated cases only took an overall proportion of 4.18% (**Table B**). Second, although the proportion was high in those aged below 30, the source of disability is predominantly attributed to diarrhea and pain in the right upper quadrant, which is quite common in those individuals with low EPG during an early stage of infection [4].

**Table B. The proportion of EPG out of range (EPG < 135 or EPG > 3525)**

| **Group** | **Proportion of EPG < 135 (%)** | **Proportion of EPG > 3525 (%)** |
| --- | --- | --- |
| **Gender** |  |  |
| Female | 9.03 | 0.00 |
| Male | 3.69 | 0.00 |
| **Age groups (years)** |  |  |
| 0–14 | 79.65 | 0.00 |
| 15–29 | 52.26 | 0.00 |
| 30–44 | 7.71 | 0.00 |
| 45–59 | 5.07 | 0.00 |
| 60+ | 5.45 | 0.00 |
| **Total** | 4.18 | 0.00 |

# Table S1: Observed and estimated EPG of the infected individuals with *Clonorchis sinensis* by provinces in China

| **Provinces** | **No. examined** | **No. positive** | **Proportion (%)** | **EPG** | **Estimated EPG (median and 95% BCI)** |
| --- | --- | --- | --- | --- | --- |
| Anhui | 91,689 | 30 | 0.03 | 267.60 | 183.63 (138.32 to 247.25) |
| Beijing | 12,537 | 0 | 0.00 | 0.00 | 309.27 (179.78 to 619.24) |
| Chongqing | 29,182 | 1 | 0.00 | 336.00 | 236.65 (166.96 to 380.48) |
| Fujian | 63,199 | 109 | 0.17 | 149.61 | 178.64 (130.59 to 261.25) |
| Gansu | 67,907 | 1 | 0.00 | 24.00 | 233.03 (136.67 to 377.45) |
| Guangdong | 108,375 | 3546 | 3.27 | 418.46 | 318.83 (278.41 to 377.65) |
| Guangxi | 73,112 | 6740 | 9.22 | 1419.96 | 441.04 (387.85 to 506.99) |
| Guizhou | 53,809 | 322 | 0.60 | 1368.82 | 813.92 (586.10 to 1158.16) |
| Hainan | 14,134 | 0 | 0.00 | 0.00 | 272.87 (168.47 to 478.67) |
| Hebei | 93,948 | 0 | 0.00 | 0.00 | 314.44 (209.69 to 486.67) |
| Heilongjiang | 86,762 | 3754 | 4.33 | 1107.82 | 368.69 (311.34 to 458.45) |
| Henan | 111,687 | 1 | 0.00 | 24,000.00 | 371.98 (241.06 to 532.20) |
| Hubei | 55,885 | 0 | 0.00 | 0.00 | 296.12 (196.84 to 470.24) |
| Hunan | 142,219 | 942 | 0.66 | 5722.20 | 741.67 (575.90 to 950.09) |
| Jiangsu | 44,392 | 18 | 0.04 | 445.50 | 202.68 (140.61 to 334.07) |
| Jiangxi | 27,515 | 112 | 0.41 | 706.50 | 166.94 (121.53 to 226.95) |
| Jilin | 76,779 | 2912 | 3.79 | 333.65 | 185.99 (159.88 to 217.70) |
| Liaoning | 82,100 | 14 | 0.02 | 5533.71 | 466.17 (258.75 to 811.22) |
| Neimenggu | 49,997 | 0 | 0.00 | 0.00 | 268.24 (194.23 to 414.94) |
| Ningxia | 21,666 | 0 | 0.00 | 0.00 | 277.06 (174.58 to 498.19) |
| Qinghai | 35,842 | 0 | 0.00 | 0.00 | 279.13 (184.32 to 507.18) |
| Shaanxi | 44,883 | 0 | 0.00 | 0.00 | 295.36 (193.81 to 455.27) |
| Shandong | 76,591 | 9 | 0.01 | 397.09 | 239.08 (169.02 to 372.90) |
| Shanghai | 11,241 | 0 | 0.00 | 0.00 | 342.74 (185.58 to 794.21) |
| Shanxi | 66,278 | 1 | 0.00 | 1848.00 | 321.38 (217.60 to 535.19) |
| Sichuan | 102,324 | 30 | 0.03 | 921.60 | 252.79 (191.73 to 354.47) |
| Tianjin | 10,098 | 0 | 0.00 | 0.00 | 318.33 (175.43 to 599.29) |
| Xinjiang | 76,995 | 6 | 0.01 | 196.00 | 238.54 (151.69 to 403.17) |
| Xizang | 20,836 | 0 | 0.00 | 0.00 | 234.23 (152.67 to 405.73) |
| Yunnan | 70,325 | 2 | 0.00 | 1296.00 | 333.00 (236.52 to 492.84) |
| Zhejiang | 51,361 | 0 | 0.00 | 0.00 | 316.35 (204.89 to 587.80) |
| **Total** | 1,873,668 | 18,550 | 0.99 | 1200.32 | 381.96 (348.39 to 419.39) |

No. examined, no. positive and proportion (%) were also demonstrated in reference [5].

# Table S2: Observed and estimated EPG of the infected individuals with *Clonorchis sinensis* by genders and age groups in China

|  | **No. examined** | **No. positive** | **Proportion (%)** | **EPG** | **Estimated EPG (median and 95% BCI)** |
| --- | --- | --- | --- | --- | --- |
| **Male** |  |  |  |  |  |
| 0–14 years | 217,581 | 358 | 0.16 | 928.06 | 231.11 (187.14 to 301.41) |
| 15–29 years | 80,538 | 660 | 0.82 | 1008.53 | 285.45 (236.10 to 360.74) |
| 30–44 years | 162,991 | 3115 | 1.91 | 1064.91 | 440.14 (371.49 to 530.95) |
| 45–59 years | 219,738 | 5107 | 2.32 | 1229.97 | 519.72 (444.77 to 628.34) |
| 60+ years | 230,911 | 2828 | 1.22 | 2034.68 | 502.84 (425.32 to 606.70) |
| Subtotal | 911,759 | 12,068 | 1.32 | 1354.87 | 435.08 (390.76 to 483.69) |
| **Female** |  |  |  |  |  |
| 0–14 years | 193,656 | 243 | 0.13 | 470.22 | 163.11 (130.26 to 212.90) |
| 15–29 years | 91,132 | 445 | 0.49 | 870.55 | 183.23 (150.26 to 232.77) |
| 30–44 years | 182,773 | 1644 | 0.90 | 743.26 | 279.57 (230.85 to 358.33) |
| 45–59 years | 247,883 | 2549 | 1.03 | 878.70 | 330.50 (280.03 to 402.40) |
| 60+ years | 246,465 | 1601 | 0.65 | 1219.17 | 328.01 (272.17 to 399.30) |
| Subtotal | 961,909 | 6482 | 0.67 | 912.57 | 282.98 (253.49 to 317.92) |
| **Total** | 1,873,668 | 18,550 | 0.99 | 1200.32 | 381.96 (348.39 to 419.39) |

No. examined, no. positive and proportion (%) were also demonstrated in reference [5].

# Table S3: Posterior summaries of the Bayesian geostatistical hurdle model parameters for *Clonorchis sinensis* intensity in term of EPG

| **Variable** | **Estimated median (95% BCI)** | **Probability (%)^a^** |
| --- | --- | --- |
| Prevalence | 0.31 (0.26 to 0.36) | > 99.99 |
| The probability of local practice of ingesting raw freshwater fish | 0.25 (0.11 to 0.39) | > 99.99 |
| Age (years) (0–14)^b^ |  |  |
| 15–29 | 0.08 (-0.07 to 0.22) | 84.60 |
| 30–44 | 0.40 (0.26 to 0.53) | > 99.99 |
| 45–59 | 0.55 (0.41 to 0.68) | > 99.99 |
| ≥ 60 | 0.52 (0.39 to 0.66) | > 99.99 |
| Gender (Female)^b^ |  |  |
| Male | 0.33 (0.26 to 0.40) | > 99.99 |
| Range for spatial effect (km) ($\rho_{1}$)^c^ | 328.70 (278.50 to 390.20) | - |
| Standard deviation for spatial effect ($\sigma_{1}$)^d^ | 4.17 (3.51 to 4.99) | - |
| Range for spatial effect (km) ($\rho_{2}$)^e^ | 102.40 (82.50 to 128.70) | - |
| Standard deviation for spatial effect ($\sigma_{2}$)^f^ | 0.83 (0.71 to 0.96) | - |
| Precision of non-spatial effect | 2.52 (2.00 to 3.15) | - |
| Scaling parameter (*β*) | -0.06 (-0.16 to 0.04) | - |

^a^Posterior probability of the coefficient > 0.

^b^In brackets, reference group is reported.

^c^The range for the spatial random effect in the model for the occurrence of local mean EPG > 0, and this spatial random effect is also shared in the model for the amount of log(EPG).

^d^The standard deviation for the spatial random effect in the model for the occurrence of local mean EPG > 0.

^e^The range for another spatial random effect in the model for the amount of log(EPG).

^f^The standard deviation for another spatial random effect in the model for the amount of log(EPG).

# Figure S1: Cross validation of the Bayesian geostatistical hurdle model for log(EPG)


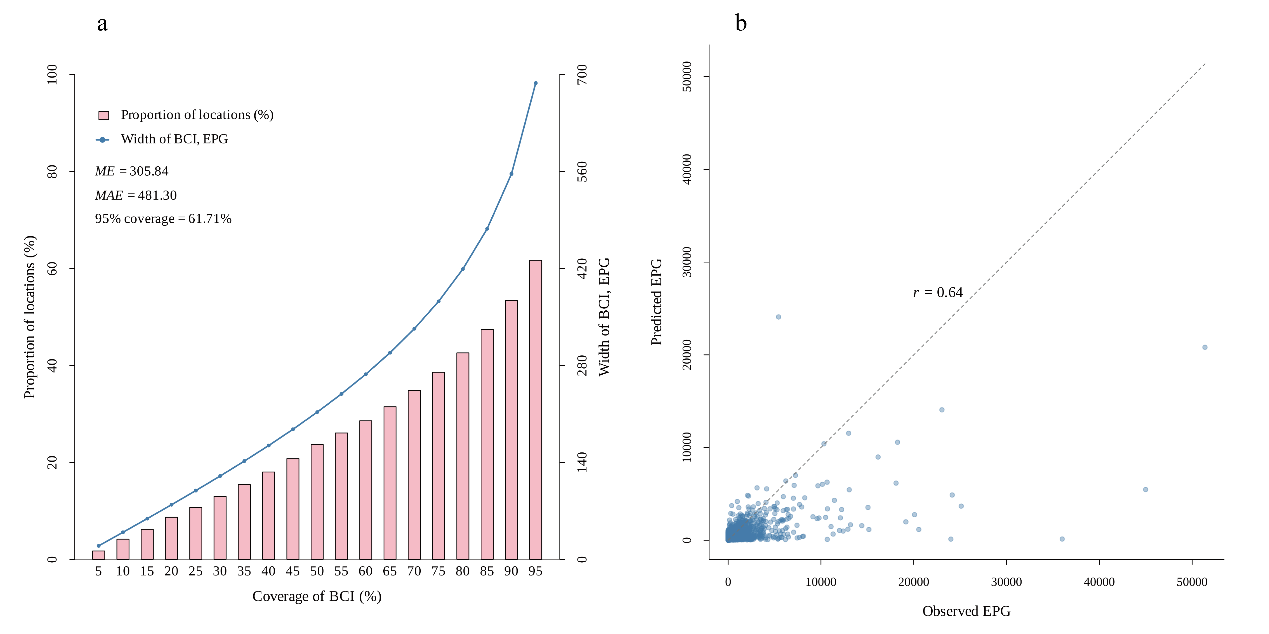


EPG represents the arithmetic mean of eggs per gram feces (EPG) in infected individuals. 5-fold cross validation was used to assess the performance of the model, with metric including (a) ME (mean error), MAE (mean absolute error), coverage (the proportion of observed values covered by the Bayesian credible interval), and (b) correlation coefficient between observed and predicted values.

# Figure S2: Cross validation of the Bayesian geostatistical linear model for log(EPG)


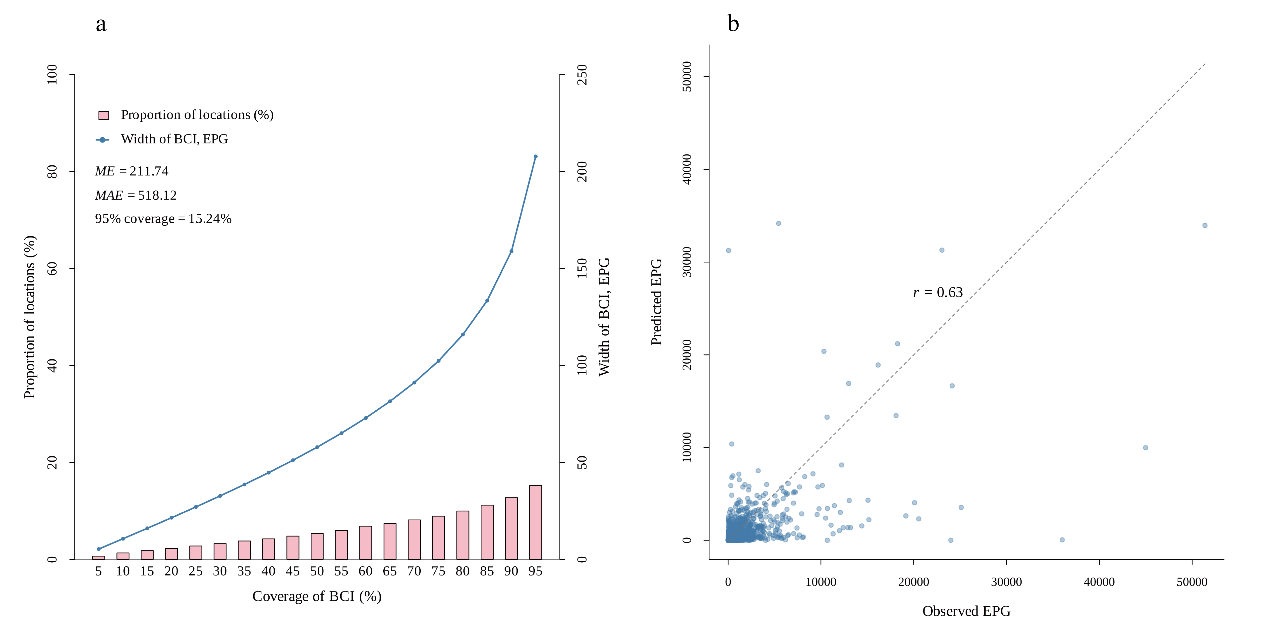


EPG represents the arithmetic mean of eggs per gram (EPG) in infected individuals. 5-fold cross validation was used to assess the performance of the model. (a) ME (mean error), MAE (mean absolute error), coverage (the proportion of observed values covered by the Bayesian credible interval). (b) Correlation coefficient between observed and predicted values.

# Figure S3: Arithmetic mean of eggs per gram feces of *Clonorchis sinensis* infection in China at 5×5 km^2^ resolution


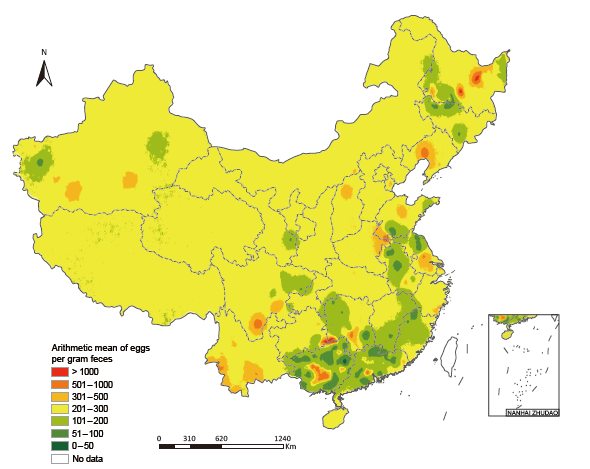


# Figure S4: Arithmetic mean of eggs per gram feces of *Clonorchis sinensis* infection by genders and age groups in China


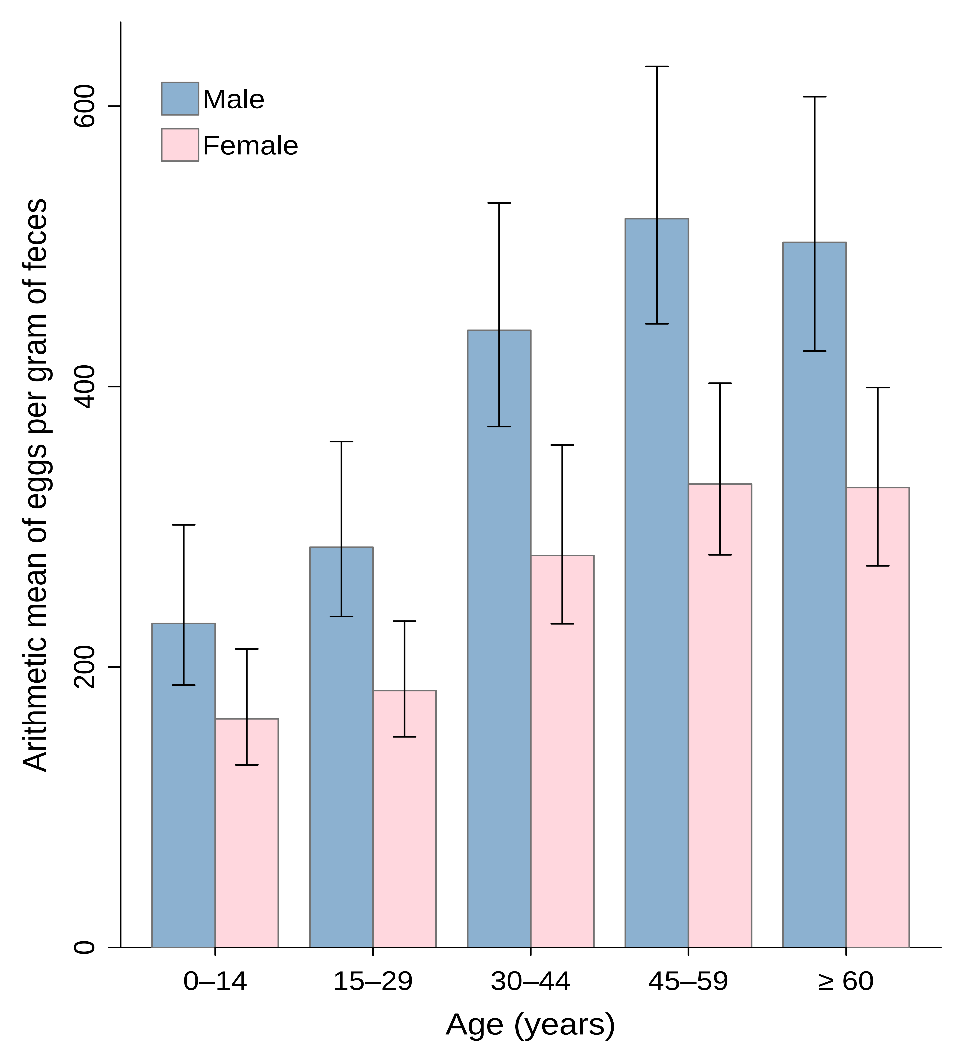


# Figure S5: Spatial clusters of *Clonorchis sinensis* infection at county level in China (disability–adjusted life years per 1000 persons)


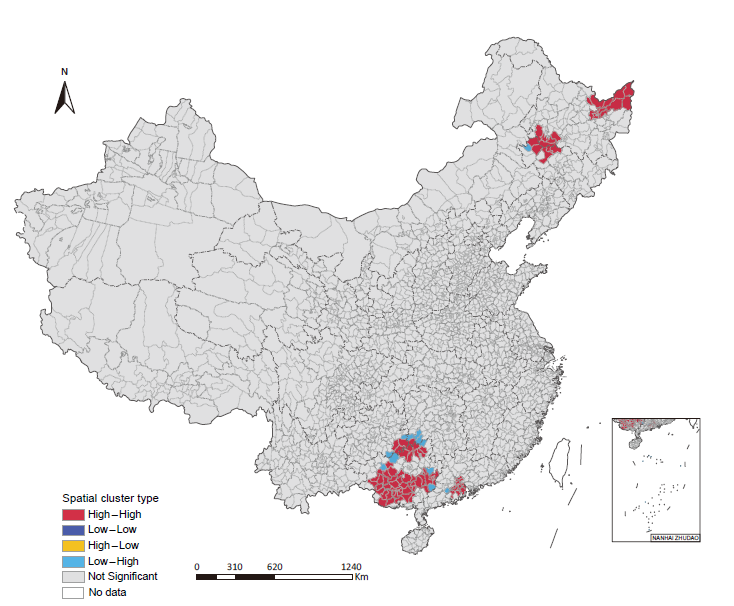


# References

1. Lindgren F, Rue H. Bayesian spatial modelling with R-INLA. Journal of statistical software. 2015;63:1-25.

2. Heilbron DC. Zero‐altered and other regression models for count data with added zeros. Biometrical Journal. 1994;36:531-47.

3. Fuglstad G-A, Simpson D, Lindgren F, Rue H. Constructing priors that penalize the complexity of Gaussian random fields. Journal of the American Statistical Association. 2019;114:445-52.

4. Qian MB, Chen YD, Fang YY, Xu LQ, Zhu TJ, Tan T, et al. Disability weight of *Clonorchis sinensis* infection: captured from community study and model simulation. PLoS Negl Trop Dis. 2011;5:e1377.

5. Qian MB, Huang JL, Wang L, Zhou CH, Zhu TJ, Zhu HH, et al. Clonorchiasis in China: geospatial modelling of the population infected or at risk, based on national surveillance. J Infect. 2025;106528.
